# Areas of Interest and Stigmatic Attitudes of the General Public in Five Relevant Medical Conditions: Thematic and Quantitative Analysis Using Twitter

**DOI:** 10.2196/14110

**Published:** 2019-05-28

**Authors:** Miguel Angel Alvarez-Mon, María Llavero-Valero, Rodrigo Sánchez-Bayona, Victor Pereira-Sanchez, Maria Vallejo-Valdivielso, Jorge Monserrat, Guillermo Lahera, Angel Asunsolo del Barco, Melchor Alvarez-Mon

**Affiliations:** 1 Department of Psychiatry Clinica Universidad de Navarra Pamplona Spain; 2 Department of Endocrinology and Nutrition Clinica Universidad de Navarra Pamplona Spain; 3 Department of Oncology Clinica Universidad de Navarra Pamplona Spain; 4 Department of Medicine and Medical specialities University of Alcala Madrid Spain; 5 Instituto Ramon y Cajal de Investigaciones Sanitarias Madrid Spain; 6 Center for Biomedical Research in the Mental Health Network Madrid Spain; 7 Department of Surgery, Medical and Social Sciences University of Alcala Madrid Spain; 8 Department of Epidemiology & Biostatistics. Graduate School of Public Health and Health Policy University of New York New York, NY United States; 9 Department of Medicine and Medical Specialities University of Alcala Madrid Spain; 10 Service of Internal Medicine, Autoimmune Diseases and Rheumatology Hospital Universitario Principe de Asturias Alcala de Henares Spain

**Keywords:** social stigma, social media, psychosis, breast cancer, HIV, dementia, public opinion, diabetes

## Abstract

**Background:**

Twitter is an indicator of real-world performance, thus, is an appropriate arena to assess the social consideration and attitudes toward psychosis.

**Objective:**

The aim of this study was to perform a mixed-methods study of the content and key metrics of tweets referring to psychosis in comparison with tweets referring to control diseases (breast cancer, diabetes, Alzheimer, and human immunodeficiency virus).

**Methods:**

Each tweet’s content was rated as nonmedical (NM: testimonies, health care products, solidarity or awareness and misuse) or medical (M: included a reference to the illness’s diagnosis, treatment, prognosis, or prevention). NM tweets were classified as positive or pejorative. We assessed the appropriateness of the medical content. The number of retweets generated and the potential reach and impact of the hashtags analyzed was also investigated.

**Results:**

We analyzed a total of 15,443 tweets: 8055 classified as NM and 7287 as M. Psychosis-related tweets (PRT) had a significantly higher frequency of misuse 33.3% (212/636) vs 1.15% (853/7419; *P*<.001) and pejorative content 36.2% (231/636) vs 11.33% (840/7419; *P*<.001). The medical content of the PRT showed the highest scientific appropriateness 100% (391/391) vs 93.66% (6030/6439; *P*<.001) and had a higher frequency of content about disease prevention. The potential reach and impact of the tweets related to psychosis were low, but they had a high retweet-to-tweet ratio.

**Conclusions:**

We show a reduced number and a different pattern of contents in tweets about psychosis compared with control diseases. PRT showed a predominance of nonmedical content with increased frequencies of misuse and pejorative tone. However, the medical content of PRT showed high scientific appropriateness aimed toward prevention.

## Introduction

Psychotic disorders are among the world’s leading causes of disability [1,2]. The estimated lifetime rate of suffering any psychotic disorder is 2 to 3% [3]. The societal and economic burden of schizophrenia is very high [4]. Schizophrenia is associated with at least 10 to 15 years of potential life lost, with no indication of a decline in this trend [5].

Despite many decades of research, the treatment of psychotic disorders remains only partially effective, and their etiology is not fully understood [6,7]. Currently, patients are encouraged to take an active role in the development of an active and meaningful life while growing beyond the misfortune of mental illness [8,9]. The traditional clinical and societal view of schizophrenia is of a debilitating and deteriorating disorder, with a poor outcome [10]. There is evidence of persisting stigma about mental illness that leads to negative stereotyping and to discriminatory behavior toward people with schizophrenia [11]. Stigma may cause affected patients to experience rejection and to feel shame about their condition, reducing their self-esteem and limiting their opportunities [12-14].

### Background

In recent years, the internet and social media have become pivotal instruments for sharing knowledge [15]. Accordingly, the internet has radically modified how most people communicate, share, and seek out information regarding health and medical conditions [16,17]. Twitter, one of the most popular and widely used platforms of social media, is currently considered an effective channel of communication [18]. Different players in health and medicine have realized Twitter’s potential for acquiring and distributing medical information [19]. Furthermore, American mainstream media outlets and the general public demonstrate a preferential interest for psychiatric disorders on Twitter [20]. A third of patients with schizophrenia use social networking sites, including Twitter, at least daily [21]. The analysis of distributed tweets is increasingly appreciated in health research [22]. The utilization of Twitter data has enabled researchers to study health-related attitudes toward behaviors and diseases, predict the incidence of both communicable and noncommunicable diseases, or have an insight of patients’ medical experience [23-25]. The utilization of online data for health care purposes has led to the development of an emerging field defined as infodemiology [26].

Moreover, the analysis of tweets about psychiatric disorders is a recent relevant area of study for understanding the sentiments of society, patients, and health players [27-33]. Concerning results have been reported about the trivialization, stigmatization, and mockery of schizophrenia and other psychiatric disorders by Twitter users [34-39]. The areas of medical and nonmedical interest of Twitter users about psychosis spectrum disorders have not been established. The reach and impact of psychosis-related tweets (PRT) remain unknown.

### Objectives

The aims of this multidisciplinary study were to investigate the medical knowledge and social consideration of Twitter users toward psychosis in comparison with 2 prevalent causes of death worldwide (breast cancer and diabetes mellitus), a relevant cause of severe neurocognitive impairment (Alzheimer disease) and a socially relevant disease (human immunodeficiency virus, HIV infection), as well as their areas of medical and nonmedical interest. In addition, we investigated the potential impact and reach derived from tweets and retweets of each condition.

## Methods

### Research Strategy

In this observational quantitative and qualitative study, we focused on searching for tweets that referred to psychosis over a period of 8 consecutive days in 2018. As controls, we studied in parallel the tweets related to breast cancer, diabetes mellitus, Alzheimer disease, and HIV infection. In this study, we focused on tweets with the following hashtags: #psychosis, #psychotic, #schizophrenic, #schizophrenia, #diabetes, #diabetic, #breastcancer, #hiv, and #alzheimer [40]. Content was limited to English-language tweets. Data collection spanned from Monday, February 26 to Monday, March 5, 2018. This period of time has at least 2 months of separation from any major international awareness month for any of the diseases we studied and was selected to avoid potential bias in the type of disease-related tweets.

### Search Tool and Data Collection

In this study, we used the Twitter Firehose data stream, which is managed by Gnip and allows access to 100% of all public tweets that match a set of “search” criteria (query) [41]. In our study, the search criteria were the previously mentioned hashtags. Tweet Binder, the search engine we employed, uses automatic machine-learning text analysis algorithms, as well as node.js and the PHP language, which enables an analysis of tweets in the json format (used by Gnip).

### Content Analysis Process

All of the collected tweets were classified using qualitative content analysis methods as a systematic method for making inferences from the text to summarize the content of communication [42]. In this study, we used a codebook specifically created by the members of the research team. All of the team’s members who qualitatively analyzed the content were medical doctors specialized in psychiatry, medical oncology, internal medicine, immunology, or endocrinology, with clinical practice in university hospitals. The analysis strategy included a series of steps. First, to achieve reliability, raters reviewed an initial subset of 100 tweets to apply initial classifications of each category. Differences in categorization and other discrepancies between the evaluators were discussed until a consensus was reached, and classification criteria were adapted to reflect the initial rating experience. Second, researchers grouped by pairs, independent and blinded, rated a second training set of 300 tweets using the improved codebook. The obtained reliability was higher than 90% for tweet content analysis, and a final version of the codebook was established. Third, all tweets were analyzed separately by 2 blinded researchers. If discrepancies in the classification of a tweet occurred between both raters (less than 10% of the cases), the whole group of researchers analyzed the tweet’s content and reached a final decision by a consensus of at least two-thirds of the research team. Tweets that included unclassifiable content were excluded.

Each tweet, depending on the content, was rated as medical or nonmedical. Medical tweets included a reference to the illness and its diagnosis, treatment, prognosis, or prevention. We also assessed if the content was medically appropriate or inappropriate according to the current medical knowledge. Nonmedical tweets were classified into 4 categories: (1) patient, family, or caregiver testimony; (2) information about medical health providers and scientific meetings; (3) solidarity, support campaigns, and advocacy; and (4) misuse. Nonmedical tweets were also classified as positive or pejorative, depending on the tone of the tweet. Classification criteria and examples of tweets by category are shown in Multimedia Appendix 1.

### Measuring Influence on Twitter: Retweets and Hashtags’ Reach and Impact

We analyzed the number of retweets generated by each tweet as an indicator of the user interest in a given topic [43-45]. We also measured the potential reach and impact of the hashtags analyzed. Impact is a numerical value representing the potential views a tweet may receive. To calculate impact, we multiplied, for each user who contributed to the hashtag, the number of followers by the number of tweets posted, and finally, we added this number for all such users. Reach is a numerical value measuring the potential audience of the hashtag (how many people could have seen it). To calculate reach, we measured the number of followers of each user who contributed to the hashtags and added them all together. We collected the 10 hashtags most frequently associated with the hashtags we studied.

### Ethical Considerations

This study received the approval of the University of Navarra Research Ethics Committee and was compliant with the research ethics principles of the Declaration of Helsinki (7th revision, 2013). However, this study did not directly involve human subjects nor include any intervention but instead used publicly available tweets. Nevertheless, we have taken care to not reveal any username and to avoid citing the tweets that could reveal it.

### Statistical Analysis

A descriptive study of the sample was performed, describing the variables by their absolute and relative frequencies. The percentages found were compared using the chi-square test. The mean numbers of retweets per original tweet about the different diseases were compared by analysis of variance. The Tamhane test was performed for a posteriori comparison between diseases.

## Results

### Increased Pejorative Sentiment and Misuse Content in Psychosis-Related Tweets

The number of tweets generated about Alzheimer disease and psychosis were lower than that of diabetes, HIV infection, or breast cancer (Table 1). Of the total of 15,443 tweets analyzed, 101 were excluded according to the criteria of the study. Thus, 15,342 tweets were classified into 2 categories according to their medical or nonmedical content, and the frequencies of both categories between the different diseases were significantly different (*P*<.001; Table 1). The percentage of PRT with nonmedical content was higher than those in the groups related to diabetes, HIV infection, or Alzheimer disease and lower than that in the breast cancer group. The percentage of tweets with medical content was higher among those related to HIV infection and diabetes than those related to the other diseases analyzed.

**Figure 1:**
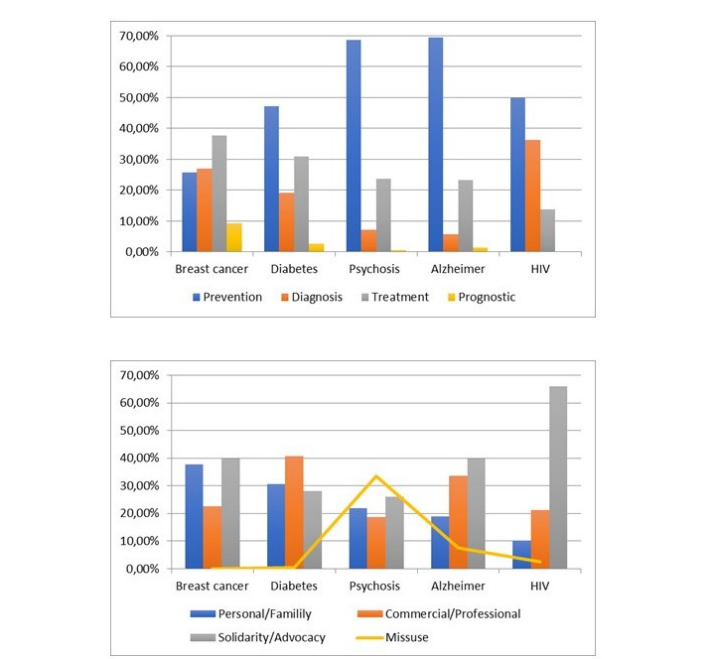
Different percentages (%) of tweets with nonmedical and medical content generated about psychosis and control diseases. Percentages (%) were calculated with respect to the total number of tweets generated about each disease.

Interestingly, different patterns of distribution of the nonmedical tweets among the 4 categorizes of contents were found between the diseases (*P*<.001; Figure 1). Remarkably, in PRT, the category with highest frequency of tweets was misuse, which was significantly higher than that found in the control diseases 33.3% (212/636) vs 1.15% (853/7419); (*P*<.001). In contrast, misuse was absent or minimal in breast cancer, diabetes, and HIV infection. The frequency of PRT with misuse content was 12 times higher than in those related to HIV infection. The frequency of tweets with solidarity and advocacy content related to HIV infection was the highest. The frequency of tweets with content about medical health providers and scientific meetings was lower in those related to psychosis, breast cancer, and HIV infection compared with Alzheimer disease and diabetes.

We analyzed the tone of the 8055 nonmedical tweets (Table 2). The frequencies of positive and nonpositive tweet contents were significantly different between the different diseases (*P*<.001). The frequency of PRT with positive content was significantly lower than what was found in the control diseases 63.7% (405/636) vs 88.67% (6522/7354; *P*<.001). The percentage of pejorative tweets related to psychosis 36.3% (231/636) doubled that of breast cancer 15.0% (365/2424) and diabetes 12.75% (677/3115) and was 5 times higher than that of Alzheimer 7.6% (38/506) and HIV 2.72% (37/1364). In psychosis and in the control diseases, the frequency of tweets with positive content was significantly lower in the misuse category than those found in the other 3 categories (*P*<.001).

**Table 1:** Number and content of tweets about psychosis and control diseases. Percentages (%) were calculated with respect to the total number of tweets generated about the 5 diseases. Number of tweets with nonmedical and medical contents generated about the diseases. Percentages (%) were calculated with respect to the total number of tweets generated about each disease.

Medical condition	Tweets generated, n (%)	Content^a^	
		Nonmedical, n (%)	Medical, n (%)
Psychosis	1029 (6.66)	636 (61.81)	393 (38.19)
Breast cancer	3703 (23.98)	2434 (65.98)	1255 (34.02)
Diabetes	6467 (41.88)	3115 (48.65)	3288 (51.35)
Alzheimer	930 (6.02)	506 (54.64)	420 (45.36)
HIV	3314 (21.46)	1364 (41.40)	1931 (58.60)
Total	15,443 (100)	8055 (52.50)	7287 (47.50)

^a^Test chi-square; *P*<.001. 101 tweets nonclassifiable (99.35% analyzed).

**Table 2:** Number of tweets with nonmedical, positive-tone content about psychosis and control diseases. Percentages (%) were calculated with respect to the total number of nonmedical contents tweets generated about each disease. Number of tweets with nonmedical, positive-tone content in the testimonies, medical health providers, solidarity/advocacy, or misuse categories generated about the diseases. Percentages (%) were calculated with respect to the total number of tweets generated about each category and disease.

Medical condition	Nonmedical content positive^a^, n (%)	Positive sentiment^b^				
		Personal/family, n (%)	Commercial/professional, n (%)	Solidarity/advocacy, n (%)	Missuse, n (%)	*P* value
Psychosis	405 (63.7)	112 (80.58)	105 (88.24)	144 (87.27)	44 (20.75)	<.001
Breast cancer	2070(85.05)	741 (80.81)	501 (91.26)	828 (85.54)	0 (0)	<.001
Diabetes	2703 (87.25)	713 (74.89)	1236 (97.86)	746 (86.04)	8 (50)	<.001
Alzheimer	457 (92.40)	94 (100)	164 (98.20)	194 (97.98)	5 (13.89)	<.001
HIV	1287 (97.28)	129 (100)	262 (100)	818 (99.63)	2 (6.45)	<.001
Total	6927 (86.70)	1789 (80.19)	2268 (96.10)	2730 (90.43)	59 (20)	<.001

^a^Test chi-square; *P*<.001. 65 tweets not classifiable.

^b^85 tweets not classifiable.

### The Medical Content of Psychosis-Related Tweets Showed High Scientific Appropriateness Geared Toward Prevention

We investigated the scientific appropriateness and areas of interest of the 7287 tweets with medical content, and we excluded 8 tweets according to the analysis criteria. The frequency of appropriateness between the 5 diseases was significantly different (*P*<.001; Table 3). According to the scientific evaluation, the content of the 391 PRT analyzed was correct. This frequency of scientific appropriateness was higher than that found in the 4 control diseases 100% (391/391) vs 93.66% (6030/6439; *P*<.001). The scientific appropriateness found in the diabetes- and HIV infection–related tweets was higher than that found in breast cancer and Alzheimer disease.

Furthermore, the tweets were classified according to the area of interest of the medical content: diagnosis, prognosis, treatment, and prevention (Figure 1). We found a different pattern of distribution of the 4 categories of medical content between tweets related to psychosis and tweets related to the control diseases (*P*<.001). Interestingly, the frequency of tweets with content about disease prevention were higher in those related to psychosis and Alzheimer disease than in those related to diabetes, HIV infection, and breast cancer. Opposite results were observed in diagnosis-related tweets. Tweets with treatment content were higher in breast cancer 37.84% (475/1255). As shown in Table 3, the lowest frequencies of scientifically appropriate medical content were found in tweets related to treatment and prognosis of Alzheimer disease.

### Psychosis-Related Tweets Showed High Frequency of Retweets

We measured the number of retweets generated about each disease (Table 4). We found that the retweet-to-tweet ratio, and thus, the probability of being retweeted for the PRT, was significantly higher than that found for the control diseases. We did not find significant differences (*P*=.49) in the frequency of retweets between those with pejorative or positive tone related to the different health conditions analyzed. We did not find significant differences in the frequency of retweets between those with misuse content and the rest of the nonmedical tweets (*P*=.08).

### Psychosis-Related Tweets Showed Limited Reach and Impact

As shown in Table 4, we found that the potential impact and reach (7,738,305 and 5,360,995, respectively) of PRT were less than those of breast cancer (62,348,473 and 20,930,244, respectively), diabetes (92,770,714 and 46,143,068, respectively), Alzheimer disease (10,019,729 and 7,118,104, respectively), and HIV infection (101,643,088 and 52,072,034, respectively). Finally, we analyzed the 10 hashtags most frequently associated with the hashtags of the different diseases analyzed. We found that the hashtags most frequently associated with #psychosis were psychosis, mentalhealth, schizophrenia, cannabis, bipolar, depression, mental illness, schoolshooting, ptsd, and wpatc18. In the case of the control diseases, the hashtags most frequently associated with #breastcancer were cancer, bcsm, mastectomy, breastreconstruction, BreastCancerAwareness, bccww, health, blog, and chemo; the hashtags most frequently associated with #diabetes were health, obesity, t1d, cancer, diet, diabetic, insulin, t2d, and type1diabetes; the hashtags most frequently associated with #Alzheimer were dementia, health, brain, care, memory, caregiver, aging, science, and alzheimers; and the hashtags most frequently associated with #HIV were aids, PrEP, stigma, USA, health, Philippines, std, vaccine, and tuberculosis.

**Table 3:** Number of tweets with medically appropriate content about psychosis and control diseases. Percentages (%) were calculated with respect to the total number of tweets generated with medical content about each disease. Number of tweets with medically appropriate content about diagnosis, treatment, prognosis, and prevention generated in the different diseases. Percentages (%) were calculated with respect to the total number of tweets generated about each different medical content and disease.

Medical condition	Medical content accuracy^a^, N (%)	Scientific accuracy^b^				
		Diagnosis, N (%)	Treatment, N (%)	Prognostic, N (%)	Prevention, N (%)	*P* value
Psychosis	391 (100)	28 (100)	91 (100)	2 (100)	264 (100)	—^c^
Breast cancer	1034 (82.52)	285 (84.07)	400 (84.39)	99 (84.62)	250 (77.40)	.05
Diabetes	3126 (95.57)	627 (100.00)	895 (88.61)	85 (97.70)	1519 (98.19)	<.001
Alzheimer	374 (89.05)	23 (95.83)	60 (61.22)	4 (66.67)	287 (98.29)	<.001
HIV	1905 (98.76)	685 (98)	258 (96.99)	2 (100)	958 (99.58)	.004
Total	6830 (94.03)	1648 (95.98)	1704 (87.88)	192 (89.72)	3278 (96.75)	<.001

^a^Test chi-square; *P*<.001. 23 Tweets not classifiable.

^b^8 tweets not classifiable.

^c^It is not possible to calculate the p value because in Psychosis the four categories (Diagnosis, Treatment, Prognosis and Prevention) had the same value (100).

**Table 4:** Potential impact, potential reach, and number of retweets generated by psychosis- and control disease–related tweets.

Medical condition	Potential impact	Potential reach	Contributors, n	Followers per contributor, n	Retweets per original tweet, mean (SE)	*P* value^a^
Psychosis	7,738,305	5,360,995	1155	19,409	0.23 (1.22)	Ref^b^
Breast cancer	62,348,473	20,930,244	3161	6621	0.03 (0.29)	<.001
Diabetes	92,770,714	46,143,068	5087	9071	0.11 (0.01)	.002
Alzheimer	10,019,729	7,118,104	1105	6442	0.04 (0.32)	<.001
HIV	101,643,088	52,072,034	7308	11,029	0.08 (0.59)	.02

^a^Analysis of variance; *P*<.001. Numbers are Tamhane test between psychosis and each disease.

^b^Ref: reference category.

## Discussion

### Principal Findings

In this work, we investigated all the tweets generated about psychosis during 8 consecutive days in the winter of 2018. As controls, we studied 2 prevalent causes of death worldwide (breast cancer and diabetes mellitus), a relevant cause of severe neurocognitive impairment (Alzheimer disease), and a socially relevant disease (HIV infection) [46]. We found a different pattern of content in tweets about psychosis with respect to those related to control diseases. PRT showed a predominance of nonmedical content with increased frequency of misuse and pejorative tone with respect to the control diseases. However, the medical content of PRT showed high scientific appropriateness geared toward prevention. The potential reach and impact of the tweets related to psychosis were low but showed a high retweet-to-tweet ratio.

The search tool utilized for data collection allows access to 100% of all public tweets. Thus, the conclusions were obtained from the results measured the *total* population of tweets, and they are not deduced from the analysis of a reduced sample (previous health-related studies utilizing Twitter have generally focused on the analysis of a 1% sample of the total number of tweets available). To our knowledge, this is the first study that analyzed *all tweets* about psychosis in particular in a defined period of time.

Our data show a differential pattern of information and opinions expressed in the contents of the PRT in comparison with those relating to the different control diseases. The majority of PRT with nonmedical content were focused on misuse, with a small proportion expressing solidarity. This bias observed in the content of PRT was further supported by the finding that more than a third of these nonmedical tweets had pejorative content about the disease and/or patients. Unfortunately, psychosis is still employed as an insult in a relevant proportion of tweets. Our ﬁndings about the elevated misuse and pejorative tone toward psychosis on Twitter are consistent with previous studies that analyzed schizophrenia in selected samples of tweets [35-38]. A recent study found that the terms psychosis/psychotic are associated with a significantly higher number of tweets with negative contents than schizophrenia/schizophrenic [34].

The relevance of this evident and extended misuse and pejorative content found in PRT is supported by the comparison of these results with those found in the investigated control diseases. Misuse in psychosis was 4 times greater than in Alzheimer disease and was marginal in breast cancer, diabetes, and HIV infection. The frequency of pejorative psychosis tweets was 5 times greater than the frequencies found in tweets related to Alzheimer disease and HIV infection and doubled those of breast cancer and diabetes. The fact that #schoolshooting was among the most frequently associated hashtags with #psychosis also reflects the negative and incorrect stereotyping of psychosis by a relevant number of Twitter users. All together, these twitter data show that psychosis patients are targets of negative ideas, feelings or judgments by twitter users, demonstrating the persistence of social stigma for psychiatric diseases, in general, and psychosis, in particular [47-50]. The bad social habit of using “schizophrenia” or “psychosis” to refer to a “madness” of some kind might have an impact on these negative results found in PRT [51]. Social stigma has major adverse effects on the lives of people with mental health conditions [52]. Stigma has also been common in portrayals of physical conditions. HIV infection has been a paradigmatic example of an organic condition suffering not only social stigma but also stigma from health care providers and professionals [53,54]. Interestingly, our results show a marginal frequency of HIV-related tweets with misuse and negativity content. We found that fewer than 3% of the HIV-related tweets had stigmatizing content, and this low number was 6 times lower than the frequency recently described [35]. This reduction may be explained by different factors, including the size and the selection criteria of the sample and the temporal gap between the 2 studies. These results support the notion of evolution in the social attitudes about diseases.

The cause of stereotypes about psychosis in Twitter is multifactorial. It may reflect the persistence of social negative stereotyping and stigmatizing attitudes toward people with psychosis [11]. Furthermore, the use of Twitter for the distribution of health care information carries some risks that are even more pronounced in the field of mental health: high rate of misinformation, sources of questionable reliability, overwhelmingly high volumes of information available, and concerns about professionalism [55]. Due to the small number of characters required, tweets are often brief and must omit key information and may lead to fruitless discussions [56]. In this context, terms such as “psychotic” or “mentally ill” can be used to disparage or ridicule someone, thus spread social stigma to the social network. Furthermore, the massive and immediate response to nonexpert opinions or news related with mental disorders can convert Twitter on an “echo chamber of ideas,” representing shared opinions rather than balanced facts because of the ease of quoting or retweeting. Furthermore, other players may lead to the production of stigma in Twitter. The public rely on the media as a key source of information about mental illness turns the news media in a strong influence on public discourse and attitudes about mental health issues as well as to medical decisions, health service utilization, or consumption of antidepressants [57,58]. Selecting the topics they cover and highlighting certain aspects, they contribute to the creation of stereotypes. It has been shown that news stories referring to mental illness frequently emphasize on violence, although rates of violence among those with mental illnesses are very low, and people with schizophrenia are more likely to be victims of violence rather than perpetrators [59,60].

Twitter appears to be a relevant communication tool for distributing and acquiring medical information. Thus, it was relevant to investigate the scientific accuracy and the areas of interest of the medical content on Twitter pertaining to psychosis and the control disorders. Interestingly, the scientific content of all the PRT was surprisingly correct, and the rate was higher than those found in the control diseases. This accuracy of tweets containing medical content starkly contrasts the high rates of misuse and negative tone in the nonmedical tweets about psychosis. These data suggest Twitter is also used to communicate medical content about psychosis by users with correct medical information. The areas of interest of the medical information varied between psychosis and control diseases. Interestingly, the frequency of tweets with content about disease prevention was markedly higher in those related to psychosis or Alzheimer disease than in those of diabetes, HIV infection, and breast cancer. Different reasons may support this differential pattern of medical interest. The social perception of limited effectiveness for the medical treatment of psychosis and Alzheimer disease might support the special interest in preventive strategies. The chronic and severe impact of patients with both diseases on their families and caregivers might also explain the high interest in the prevention of these disorders [10,61,62]. The absence of established analytical or image criteria for the diagnosis of psychosis might also contribute to the limited interest in the diagnosis of the disease.

Finally, we investigated the interest and diffusion of the tweets generated about psychosis. In the period of time analyzed, the frequency of retweets generated by PRT was higher than that found in the control diseases. This parameter is considered an indicator of the user interest in the topic of each tweet [43-45]. However, the metrics of the tweets related to psychosis were small compared with those of the control diseases. The impact of the PRT measured as the potential views that the tweet may receive was clearly lower than that of the control diseases. Similarly, poor diffusion of the PRT was found when we calculated the potential audience or reach of the PRT. These metrics are used for the quantification of the diffusion of tweets and potential influence in society. Thus, our data imply that the stigmatization of and limited social support for psychosis are reflected in the low impact and reach of the PRT.

### Strengths and Limitations

Although this study improves and expands previous research on the communication of psychosis in a popular and widely used form of social media, there are still some limitations. The rating process had an inherent degree of subjectivity because of differences in the perceived context and emotional tone of some tweets. This was made particularly evident by words that had dual meanings. There was also a degree of selection bias, as stigmatizing and trivializing tweets were more likely to be lacking in context and/or grammatical correctness, rendering them less likely to be considered for analysis. We minimized the effects of these issues through our robust rating criteria and binary rating system, which were chosen for the analysis performed by expert clinicians in the medical fields analyzed. To achieve maximum reliability, the qualitative analysis of the disease-related tweets required a manual input that was time-consuming and required expert involvement. We followed what we considered a gold-standard qualitative analysis strategy.

### Conclusions

Twitter is a tool for developing interventions and strategies of information aimed at modifying health-related social and individual behaviors [63]. Our results support the dynamic and potentially positive evolution of the social stigmatization of health disorders, as can be observed in HIV infection and cancer. Thus, it is possible to expect a reduction of psychosis stigmatization. Moreover, mixed-methods research on Twitter and other social media may be a relevant strategy for measuring the effectiveness of the strategies and actions established for overcoming the social psychosis stigma [64]. A proactive sensitization by professionals, scientific societies, patient associations, and other social agents to use the promising platform of social media communication is needed. Furthermore, the respect and supportive in social media communication content may also impact a patient’s life and treatment. Psychosis patients often use social media [21], and despite the anonymity of Twitter, many users identify themselves as patients [27,32].

Although stigmatization is significantly decreasing and societal consideration is improving in other disorders, such as breast cancer and HIV, the stigma regarding psychosis is not decreasing. On one hand, psychosis is used as hate-speech on Twitter, but on the other hand, Twitter is used as a beacon of medically accurate information for the disorder. Therefore, Twitter may be a great tool for antistigma campaigns and promotion of healthy habits.

## Appendix

Multimedia Appendix 1 Classification criteria and examples of tweets by category.

## Abbreviations

PRT: psychosis-related tweets
