# Supplementary material for: Areas of Interest and Stigmatic Attitudes of the General Public in Five Relevant Medical Conditions: Thematic and Quantitative Analysis Using Twitter
Source: J Med Internet Res. 2019 May 28;21(5):e14110. doi: 10.2196/14110 (PMC6658306; doi:10.2196/14110)
Supplement: Multimedia Appendix 1 [file jmir_v21i5e14110_app1.docx]

Classification criteria and examples of tweets by categorie.

| **Diagnosis:** Diagnostic tests or epidemiological data | - Per Time Magazine, "1 in 10 people in the U.S. over age 65 has Alzheimer's. By 2050, without effective treatment, 16 million could be affected by this disease. Worldwide about 50 million people have dementia." #Alzheimer's, #Dementia, #Eldercare, #Caregiver, #Caregiving |
| --- | --- |
|  | - Hallucinations and paranoia can occur in very extreme cases of depression. Please if you notice someone displaying these symptoms, kindly refer them to a specialist for further evaluation. #Depression #Psychosis |
|  | - Constant thirst, tiredness and urinating a lot are the main symptoms of #diabetes. See your GP if you're worried https://t.co/SYrQu2OwX7 https://t.co/RrqyUhzaCI #ExtremeCasesOfDepression #LetsTalk #FightingDepressionWithSEF https://t.co/gfG45s9fA1 |
|  | **-** Zambian Peer Educators for HIV Self-Testing (ZEST) study: Rationale & design of a cluster randomised trial of #HIV self-testing among female #SexWorkers in #Zambia https://t.co/sOjCEbGHhB HT @HarvardChanSPH #SexWork #FSW #SexWorker |
|  | - #Schizophrenia impacts about 1% of the world. See the findings of a recent study on this #disorder: https://t.co/MsguGzsTo8 https://t.co/ZjPVIrL1TP |
| **Treatment:** New drugs or side effects. | **-** #Marijuana Removes Toxic #Alzheimer’s #Protein from the #Brain via PotMedsMinder app for tracking and logging #meds #marijuana for #medical users professionals. |
|  | - Typically there are long delays in commencing clozapine in those with #psychosis with persistent symptoms - Dr Kristen Thien found approx 10% of young people with early psychosis will be eligible to commence clozapine #WPATC18 https://t.co/wRO4294Aaa |
|  | - Swedish study showing that use of antipsychotics and particularly 2nd generation LAI antipsychotics lowers mortality in schizophrenia and first Episode Psychosis. Avoid benzodiazepines use more than one month....confirms clinical practice.... #EPA2018 #psychosis |
|  | - NICE JOB @Flinders Eye+Vision Health Dr. @binoyappukuttan: 2nd paper published in past 3 months = Translational corroboration of studies in experimental models indicates @Genkyotex_Off Nox1/4 inhibitor may help |
|  | - Article online: phase 3 trial of #adjuvant anastrozole vs exemestane vs letrozole, upfront or after 2 years of #tamoxifen, in endocrine-sensitive #breastcancer (FATA-GIM3 study) https://t.co/bZm6LaC8Fh |
|  | - European Panel Recommends Against Neratinib for HER2-Positive #BreastCancer https://t.co/i0AlL23GxD via @onclive #bcsm |
| **Prognosis** | **-** Long-term mortality investigated in all weight categories in type 2 diabetes https://t.co/o7QYgpjM8B via @Diabetescouk #Health #Diabetes #insulin #weight #twitter #truth |
|  | - New study from @dynahealthEU @Folkhalsan researchers shows effective treatment and early diagnosis of #diabetes means individuals exited workforce two years later than those without diabetes |
| **Prevention:** Health promotion. | - Even slight elevations in blood sugar have been shown to increase the risk of #Alzheimer's disease. |
|  | - Discover the Difference Between #Alzheimer’s Disease, #Aging, And #Dementia |
|  | **-** #Alzheimer's disease, the most common form of #dementia, is characterized by the accumulation of two types of protein in the brain: tangles (tau) and plaques (amyloid-beta). |
|  | **-** Increasing physical activity 🏃‍♂️🚴‍♀️🤸‍♂️ is a key element in controlling weight & reducing likelihood of developing type 2 #diabetes. |
|  | - Snow permitting we'll be making our way to @onlyathalfway later this afternoon. We'll be providing rapid #HIV testing, #free condoms, #sexualhealth and #PrEP information and advice. More details @ https://t.co/zAztptc4Om #gettested and #knowyourstatus https://t.co/KGakZKNhH4. |
| **Patient, family or caregiver testimony:** Sharing experiences | - #diabetes #diabetic #t1d TRYING THE OMNIPOD FOR THE FIRST TIME! https://t.co/ib9MLRuH2c #type1diabetes #diabetes #t1d |
|  | - Cancer I could deal with. Losing my breast I could not: https://t.co/XNc5vuN6Zs #BreastCancer |
|  | - On being overwhelmed and so damn grateful at the same time; glad to be alive glad the #breastcancer was found and could hopefully be cured and mostly thankful for my Sherpa who taught me all I needed to know to survive it and trying to be that for others https://t.co/IxcYn5inNK https://t.co/1TKe1n0gxi |
|  | - Writer & #HIV long-term survivor Randy Boyd shares his journey so far in a new collection of past writings, THE ESSENTIAL RANDY BOYD. Interview by @ChaelNeedle Photos by @seanblackphoto https://t.co/PsBwbNrqjy @randyboydauthor https://t.co/i9mieqjDU0 |
|  | - "Paula Lovely is my drag persona" – Paolo tells his #HIV story. Watch his interview and 10 others stories here https://t.co/JCZdcukPRx #zerodiscrimination #UNAIDS #HIVpositive #HIVisjustapartofme https://t.co/xqaOqFd2NP |
|  | - Yesterday my #Yorkie #Martini was diagnosed as #diabetic. Injections 2x a day. Mentally challenging, but he's doing really well. 💙 https://t.co/o5T0h1EHyl |
|  | - #Alzheimer's I have just published Mangrove Haiku, a book about my relationship with my father-in-law who had Alzheimer's and me. In the wetland mangrove, we found metaphors and reasons to live. My journey with him renewed my intention to live life. |
|  | - Veteran #Caregiver for #MultipleElders Tells It Like It Is https://t.co/xm5aPduGRd #Alzheimer's via @HealthCentral #dementia #aging #caregiving #eldercare. |
| **Medical health providers, scientific meetings or awards:** Reference to or advertising for a service or product for sale. Information from a congress, scientific meeting or interview/meeting with an expert | - Diabetes damages essential systems in your body: your blood vessels, nerves, or both. The consequences of uncontrolled diabetes can be very serious.  Call 85-888-56-222 for FREE #Diabetes Consultation and Ayurvedic Medicine of Diabetes. https://t.co/Q6yr1pbOSe #diabonashak https://t.co/aVs1Kd8u9h |
|  | - What if these products could change your life? Take the step and order today. I will help you on a road to better health. Commit 90 days to your |
|  | - Experience the advanced healthcare, with customized diabetes care. Experience #PrecisionDiabetes only at Dr. Mohans’s Diabetes Specialties Centre. For Appointments: https://t.co/TAU2JgUrwb #DMDSC #Diabetes https://t.co/7oyV3QizQw health! Message me today free gift with order. https://t.co/ZJGC8eBTx2 #Supplement #diabetes #immune #weightloss #wellness https://t.co/fR6Cuo7aE1 |
|  | - A yearly mammogram is the most effective way to prevent early breast cancer deaths. At @brexahealth, we offer mammogram vouchers at the discounted rate of Rs 1500. Buy yours: https://t.co/0u8NYtdABD #breastcancer #breastcancerawareness #mammogram #knowyourrrisk #BCSM #BCWWW https://t.co/1WuYypqFPi |
|  | - @__kld__ Hi there! On our website! 😁 Here is the purchase link for you: https://t.co/H1Hrlc1KcG remember to tick the vat exemption box so it’s £44.99 if you are a #Breastcancer or #Cardio patient in recovery ☺️ |
|  | - Got frisky? Was it risky? Find the #HIV test that suits you best! https://t.co/rTv4IrKoVC |
|  | - Looking forward to an exciting @AlzheimerEurope #AELunchDebate in #Brussels @Europarl_EN tomorrow. |
|  | - Accelsiors’ team will be joining scientific exchanges on all aspects of breast cancer clinical trials at the EBCC-11 meeting. Join us in Barcelona, 21-23 March 2018: https://t.co/l0odEIxM6t |
|  | - Have your thumbs ready for our special forum discussion sessions! You will have the opportunity to ask your questions via the Ask the Speaker function in the event mobile App. |
|  | - Save the date and join us on Tues, Mar 27, for the RECEPTION honoring the 2018 winners of the Rosalinde Gilbert Innovations in #Alzheimer’s Disease Caregiving Legacy Awards during #AiA18: https://t.co/MTcP1A7y4n @ARTZPhilly @jfcsboston @LREcares @ASAging https://t.co/hRILmvtpPv. |
|  | - Looking forward to #ADEA2018: "#Screening for #Diabetes: The @UMSOD_Dentistry Experience" https://t.co/8zED2bOww6. "Devices to Screen/Monitor for #Diabetes" https://t.co/gBE8kr9UX0 @UMBaltimore |
|  | - Happy to be part of the panel speakers @MUSChealth Global Health Week 'Our World,Our Health @NACC_Kenya @NASCOP #HealthyNation #HIV #football @MaishaYouth https://t.co/uSpqMmzs1V |
|  | - @NIH will host this year’s #Alzheimer’s Disease Research Summit from March 1-2 at the NIH Campus in Bethesda, MD. Find more info about the summit here: https://t.co/bJPZtJ1pFa |
| **Solidarity, awareness campaigns or patient advocacy:** Messages of hope or support; combating stigma; raising awareness (not including scientific data); fighting and campaigning; giving people a voice; connecting or socializing with other users; sharing or receiving information | **-** Today is CA #Alzheimer’s Advocacy Day in Sacramento! Looking forward to seeing all my purple peeps & asking #CAleg to #Act4Alz! |
|  | - 10 Easy ways to make your home Dementia friendly #Alzheimer'sSociety #DementiaFriends https://t.co/Y7ewy5mjMp. |
|  | - “I decided to come to Africa because I had accomplished everything in America, people #diabetes needed me more in Kenya” Jean Soren @angelamwihaki #BreakfastKBC https://t.co/gYrrENJYvo |
|  | - We’re looking for parents of children with #diabetes to help us make information about the care they receive easier to understand. Find out more: https://t.co/wytnK6bf5C https://t.co/Hs5eWlyWv3 |
|  | - I have just signed up for the Mother's Day Classic fun run that will raise money to continue to fund the much needed research in this disease. Any donation gratefully received. https://t.co/y2BwQHunBp #breastcancer |
|  | - Congrats to @JDRF on their One Walk which has raised more than $1.5m for Type 1 #diabetes research 🙌 read more here here: https://t.co/wAaZreNKyY |
|  | - Play for PINK golf tournament benefits breast cancer research - Sun Sentinel https://t.co/aWojJUQwZ0 #breastcancer https://t.co/hw4WjAufCy |
|  | - Who will you Drink Pink for!? #awarenesscoffee #spreadtheword #coffeeforacause #drinkpink #awareness #breastcancer @NBCF @YSCBuzz @WilmotCancer https://t.co/k2rVgRZCgm |
|  | - Today we mark #WorldNGODay, we stand in solidarity with our fellow #NGOs all around the world. As we fight #BreastCancer, #CervicalCancer and #ProstateCancer @kenconetwork #CancerFeeGeneration https://t.co/2qyIUyZVNO |
|  | - Into action ! RT #WomensHistoryMonth #Jakarta #Indonesia #HumanRights #MomensMarch #NoStigma #SupportDontPunish #HIV #aids2018 #harmreduction https://t.co/4hgsc5KOLx |
|  | - March is #WomensHistoryMonth! NASTAD recognizes the many contributions women have made throughout history, particularly those who have championed the fight against #HIV, #hepatitis, and injustice. We celebrate and salute you. https://t.co/DeIFpoT6JV |
|  | - What it means to be "positive" or how to #think post #cancer diagnosis https://t.co/QMxN6GeDOt #breastcancer #fighter #tips |
|  | - When undergoing treatment for #breastcancer, imperative to include your #mentalhealth. Here's a list of resources: https://t.co/TPDYGz5f4i #chemotherapy #radiation #BRCA #TNBC #selfcare #thriver #OurTribe |
|  | - When every day is the same old boring 💩 of tiredness, boredom, nausea, frustration and isolation. #chemo treatment really is getting on my wires! Need normality and the daily routine back not stuck in the house 80% of the time 😠😠 #fuckcancer #breastcancer |
|  | - To stay up to date on the latest info. on #breastcancer follow @brstcancerchat & join their monthly #twitterchat #share #RT #socialmedia |
|  | - From cold caps to constipation, no topic is off limits when it comes to talking about #breastcancer side effects on our discussion boards #bcsm https://t.co/J01OFdOib8 https://t.co/76mDo04MMf |
|  | - #Porsha "i clearly have raw sex you guys" #RHOA UMMMMMMMMMM ima need you to elaborate on that.... have your partners or partner been tested for #HIV among all the rest of the #STDs #STIs lurking in peoples #genitalia? #QTNA #FACTS #RHOA |
|  | - is there any reality to the mental illnesses or its just an illusion to get away from your dark side? #mentalhealth #bipolar2 #BPD #ADD #Schizophrenic |
| **Misuse:** Mockery; glamorization; trivialization; using the illness as a compliment or an insult; wishing harm upon someone by way of contracting the target condition; associating the illness with undesirable attributes; demeaning the target condition by joking about it; associating the illness with grossly inaccurate stereotypes | - Are you fucking kidding? How the fuck is this News? No wonder the US crumbling. You think #DonTheCon 卐 #OrangeHitler 💩4🧠卐 #DotardDrumpf. #SENILE #Alzheimer #ILLITERATE #OBESE #BALD. Understand Finances, yet alone Global FINANCES? Are you fucking CRAZY? https://t.co/1UyHeVlLia. |
|  | **-** Cancer PTSD: Help From a Breast Cancer Survivor https://t.co/3U0fOK5VkY #hyperbaricchamber #hyperbaric #veterans #autism #alzheimer #ptsd #stroke #dementia #hbot #success #recovery #oxygen #strokerecovery #lymedisease #oxygentherapy #health #pain #fatigue #menshealth #DoctorLou |
|  | - Since he wants to lock up the mentally ill, we need to start w/ #Trump. Trump is a #psychopath, suffers #psychosis and is an 'enormous present danger', says #psychiatrist \| The Independent https://t.co/ctr87GB6W9 |
|  | - @GiffordsCourage You want to fight gun violence? Go to #Chicago #Baltimore #East St. Louis #Detroit #Washington D.C. and get rid of the #black #Caucasus who have ran these cities for the last 60 years. #liberalism is an #epidemic of #psychosis. #nra #MAGA |
|  | - @HuffPost Trump is #schizophrenic. Will @GOP not be satisfied until this aging narcissist ruins our world stature and economy because of a tax cut and gun rights? #PaulRyanMustGo #MitchMcConnell is challenging #TedCruz for the title of Lucifer in the flesh. |
|  | - Money is like #HIV when you ask, Everyone says they don't have it. #Goodmorning.... #MondayMotivation. #Joke https://t.co/ErIb6jKq2P |
|  | - @neeratanden Just imagine #kushner and #Ivanka. Again @realDonaldTrump is a #schizophrenic #narcissist #traitor #racist #bigot #liar #cheater #bully #insecure and very #dumb #loser |
